# Tuning Apparent Peak Efficiency in Capillary Electrophoresis Using Backscatter Interferometry Detection

**DOI:** 10.1002/elps.70059

**Published:** 2025-12-06

**Authors:** Miyuru De Silva, Stanslaus M. Kariuki, Robert C. Dunn

**Affiliations:** Department of Chemistry, Ralph N. Adams Institute for Bioanalytical Chemistry, University of Kansas, Lawrence, Kansas, USA

**Keywords:** capillary electrophoresis, backscatter interferometry, peak efficiency, resolution

## Abstract

Backscatter interferometry (BSI) is a refractive index detection method for capillary electrophoresis that is inexpensive, flexible, and easily miniaturized. Interestingly, unlike most detectors that respond exclusively to analyte concentration, the BSI signal is sensitive to both refractive index (analyte concentration) and the separation voltage. The latter is linked to zone conductivity and leads to improved BSI signals and lower detection limits with increasing field strengths. Enhanced BSI signals can also be generated using a photothermal mechanism, where resonantly excited analytes release heat into their surroundings to increase the BSI signal amplitude. Both voltage-based and photothermal signal enhancement mechanisms can lead to a change in the polarity of the BSI signal, which can be either positive or negative depending on the specific analyte, its concentration, and the separation conditions. Here, we show that this leads to a significant increase in apparent peak efficiency. At the transition in peak polarity, both mechanisms result in over a 10-fold increase in apparent peak efficiency, improving from approximately 10^5^ plates/m to over a million plates/m. Simultaneously measured BSI and fluorescence electropherograms confirm that the efficiency increase is unique to the BSI signal and not due to changes in zone dispersion, and can be tuned to optimize separation resolution. The origin of the efficiency increase is discussed in terms of the refractive index and zone conductivity contributions to the BSI signal.

## 1 | Introduction

Analytical separations are essential in chemical analysis, and a myriad of chromatographic and electrophoretic separation approaches have been introduced. Despite operating under different principles, all methods share the same objective of separating and detecting constituents in complex sample mixtures. The separated species are typically detected as peaks, and for accurate qualitative and quantitative analysis, the experimental conditions must be optimized so that the peaks are sufficiently resolved from one another. To evaluate the effectiveness of a separation, parameters such as resolution and peak efficiency are often quantified.

The resolution, *R*, describes the degree of separation between two analyte peaks and is given by Equation (1):

(1) $$R=\frac{2\left(t_{2}-t_{1}\right)}{w_{1}+w_{2}},$$

where *t*_1_ and *t*_2_ are the migration times of the two peaks (*t*_2_ > *t*_1_) and *w*_1_ and *w*_2_ are their respective baseline widths in units of time [1]. As shown in Equation (1), resolution improves as peaks narrow. The narrowness of a peak is quantified by peak efficiency or plate number. Initially used to measure the efficiency of distillation processes, theoretical plates or efficiency was adapted to characterize Craig countercurrent separations [2], chromatographic separations [3], and later capillary electrophoresis (CE) [4]. Plate number or efficiency is particularly useful for comparing different separation techniques and instruments. Assuming Gaussian-shaped peaks, the number of theoretical plates can be calculated using Equation (2):

(2) $$N={\left(\frac{l}{\sigma }\right)}^{2}=16{\left(\frac{t_{m}}{w}\right)}^{2}=5.54{\left(\frac{t_{m}}{w_{0.5}}\right)}^{2},$$

where *l* is the length-to-detector, *σ* is the standard deviation of the peak, *t_m_* is the migration time, *w* is the baseline peak width, and *w*_0.5_ is the peak full-width at half-maximum [5]. As shown in Equation (2), narrow peaks are associated with large plate numbers.

A distinct advantage of CE over conventional chromatographic techniques is the high efficiency of the technique. This arises from the lack of a stationary phase, and the subsequent band broadening that results from mass transfer, and the plug-like flow profile in electrophoretic separations versus the laminar profile of pressure-driven techniques [6, 7]. Because of these factors, the efficiency in CE is limited only by longitudinal diffusion and can routinely achieve efficiencies of hundreds of thousands of theoretical plates per meter [8].

While longitudinal diffusion represents the theoretical limit on efficiency, several mechanisms can contribute to band broadening in real-world CE applications. During the separation process, any mechanism that broadens the velocity profile of an analyte will lower the efficiency. This can include mechanisms such as analyte adsorption to the capillary walls [9, 10], electromigration dispersion arising from conductivity differences between analyte and background electrolyte (BGE) [11], and Joule heating which creates temperature gradients in the BGE [12]. Unwanted band broadening can also result from the specifics of the instrumental approach. For example, uneven buffer levels at the inlet and outlet of the capillary can lead to siphoning, creating a laminar flow profile that lowers efficiency, or the detection method itself can broaden measured bands if the detection zone is larger than the length of the analyte zone [13, 14]. These are all well-known broadening mechanisms, and optimization strategies have been developed to reduce their influence [15–17].

For challenging separations of analytes with very similar electrophoretic mobilities, such as isomers, resolution is normally improved by increasing the capillary length or by reducing or reversing the electroosmotic flow [18, 19]. While both strategies are effective, they can significantly increase analysis time. Alternatively, the link between resolution and plate number (*N*) has led to the development of various stacking techniques to compress analyte zones and enhance *N* [20, 21]. Here we offer an alternative approach that enhances the “apparent peak efficiency” and, consequently, the resolution between peaks in capillary electrophoresis. Unlike methods that seek to reduce zone dispersion, this approach leverages the unique features of the backscatter interferometry (BSI) signal to narrow the measured response from analyte zones [22, 23].

BSI detection uses the interference fringe pattern created by coherent light focused into a separation capillary to detect analyte zones based on refractive index (RI) changes [17, 22, 24–27]. Zones passing through the detection volume alter the RI and optical pathlength, shifting the fringe pattern, which is detected as the BSI signal. In general, RI signals have relatively poor detection limits, which is exacerbated in CE by the short path length of the narrow capillaries. Considerable effort, therefore, has been devoted to developing methods that enhance the signals and improve detection limits. Our group, for example, has explored methods based on the separation voltage and photothermal excitation to improve detection limits in BSI [28, 29].

The BSI signal responds to both the RI and the separation voltage [17, 28]. For the latter, the BSI signal amplitude increases linearly with the square of the separation voltage [17]. A model was introduced that showed the RI-dependent component governs the signal at low separation voltages, while the voltage-dependent component grows and eventually dominates the BSI signal at high field strengths [28]. The latter arises from the conductivity difference between the analyte zone and the BGE, making the BSI detector similar to a conductivity detector at high field strengths. In this regime, there is a linear increase in peak area with separation voltage, providing a useful avenue for improving detection limits [28].

Another method for improving BSI detection limits involves photothermal excitation [29–34]. In this approach, analytes are resonantly excited either with the BSI laser itself or a second laser beam directed coaxially through the same detection volume [29]. Nonradiative relaxation of the excited analyte releases heat into the local surroundings, which increases the temperature-sensitive RI signal and improves detection limits by orders of magnitude [29].

Analyte peaks detected by BSI can be either positive or negative depending on the specific analyte, its concentration, the BGE, and separation conditions. In both the voltage-based and photothermal enhancement methods, peaks can change polarity during the enhancement process, which leads to an interesting narrowing effect at the transition. This narrowing effect is explored here as a mechanism for increasing the “apparent peak efficiency” to improve separation resolution. Specifically, we show that BSI detection combined with either electric field enhancement [28] or photothermal enhancement [29] can improve the measured peak efficiency by over an order of magnitude, even though the zone dispersion remains unchanged. This approach is inspired by advances in other areas where optical signals are manipulated to improve performance [35–38]. We show that careful tuning by either the separation voltage or photothermal enhancement mechanism can optimize apparent peak efficiency in the BSI signal and lead to improved resolution for closely migrating analyte zones. While the voltage-dependent mechanism is unique to CE, the photothermal scheme should be operable in chromatographic separations as well.

## 2 | Materials and Methods

### 2.1 | Chemicals

Arginine, lysine, leucine, and isoleucine were obtained from Sigma-Aldrich (St. Louis, MO). Reagent-grade sodium chloride, potassium chloride, lithium chloride, imidazole, rhodamine 123(R123), rhodamine 6G (R6G), and acetic acid were acquired from Fisher Scientific (Hampton, NH). All chemicals were used without further purification. Stock solutions of the amino acids and dyes were prepared at concentrations of 1 mM and refrigerated at 4°C before use. To prepare samples, stock solutions were diluted to the desired concentration and allowed to warm to room temperature. Acetic acid and imidazole BGE solutions were prepared at the indicated concentrations, and their pH was adjusted with the addition of hydrochloric acid. All solutions were prepared using ultrapure water and passed through a 0.22 μm filter (Fisher Scientific, New Jersey) before use.

### 2.2 | Instrumentation

The home-built planar capillary electrophoresis (PCE) instrument has been described previously [17, 23]. Briefly, a 10 cm long fused silica separation capillary (50 μm i.d. × 80 μm o.d.; VitroCom, Mountain Lakes, NJ) is mounted flush on a heat sink and its length covered with a thermal paste (SILV5, StarTech), except for the ends and detection zone. The outlet of the capillary is sealed against an O-ring enclosure to enable an under-pressure for fluid exchanges and capillary conditioning. The capillary inlet is positioned above small vials holding sample and run solutions that are affixed to a computer-controlled rotating platform that positions the appropriate vial at the capillary inlet. All separations were run in normal polarity using a separation voltage supplied by a Spellman CZE 2000 high-voltage power supply. The positive potential was applied to a platinum electrode immersed in the BGE at the capillary inlet while the outlet reservoir was grounded.

The PCE platform is mounted on an inverted fluorescence microscope (Olympus IX71) with the capillary detection window (8 cm length-to-detector) centered above the microscope objective. For BSI detection, the appropriate laser line is coupled into a single-mode optical fiber, which is positioned above the capillary. Light exiting the fiber is focused into the capillary, and the backscattered interference pattern is directed toward a bicell photodiode detector (EG&G Optoelectronics, Quebec, Canada). Two fringes in the backscattered interference pattern are aligned on a segmented photodiode detector and differentially amplified (Stanford Research Systems, SR560) to form the BSI signal as discussed previously [17, 23]. For photothermal excitation, the appropriate laser line is coupled into the same optical fiber used to deliver the BSI laser line, leading to coaxial excitation in the same detection volume [29]. For fluorescence detection, sample fluorescence is collected from below the capillary with the microscope objective (Nikon 10x, 0.30 NA), filtered to remove excitation light (Chroma Technology, Vermont, USA), and detected using a photomultiplier tube (PTI 01-612, Photon Technology International).

BSI electropherograms of amino acids were measured using the 647 nm line of an argon-krypton laser (Coherent Innova 70, Santa Clara, CA). The remaining studies used either the 543 nm line or the 633 nm line from HeNe lasers (Research Electro-Optics Inc., Boulder, CO) for BSI detection, as indicated. For the photothermal studies, the 476.5 and 488 nm lines of an argon ion laser (Coherent Innova 90) and the 532 nm line from a diode laser (ThorLabs, Newton, NJ) were utilized as indicated.

### 2.3 | Experimental Procedures

Before separations, the PCE capillary was flushed with conditioning solutions by positioning the appropriate solution at the capillary inlet and applying an under-pressure at the capillary outlet. The capillary was flushed with 1 M NaOH for 10 min, rinsed with nanopure water for 5 min, and conditioned with the appropriate BGE for 10 min. Amino acid samples were injected hydrodynamically using an underpressure of 5 kPa for 1 s. Experiments were repeated three times unless otherwise noted. Dye-containing samples were electrokinetically injected onto the capillary at 5 kV for 1 s. Signal collection and instrument control signals are routed through a data acquisition module (National Instruments, USB-6001) using custom-written software in LabVIEW (National Instruments). Data processing was performed using Origin (OriginLab Corporation) and Microsoft Excel software packages. Peak efficiencies were calculated using the open-source CEval software package that uses the Haarhoff-van der Linde function for analyzing triangular-shaped peaks [39].

## 3 | Results and Discussion

### 3.1 | BSI Signal Polarity

When using conventional detection methods like UV–Vis or fluorescence in CE, the separated zones are measured as peaks that reflect the concentration profile of analytes as they migrate past the detection zone. Ideally, this distribution follows a Gaussian shape due to diffusion at the zone boundaries. Factors such as electromigration dispersion, however, can lead to non-Gaussian, triangular-shaped peaks. Regardless of the peak shape, each point measured along the peak profile is determined by the local concentration of the analyte at that point.

**FIGURE 1 |:**
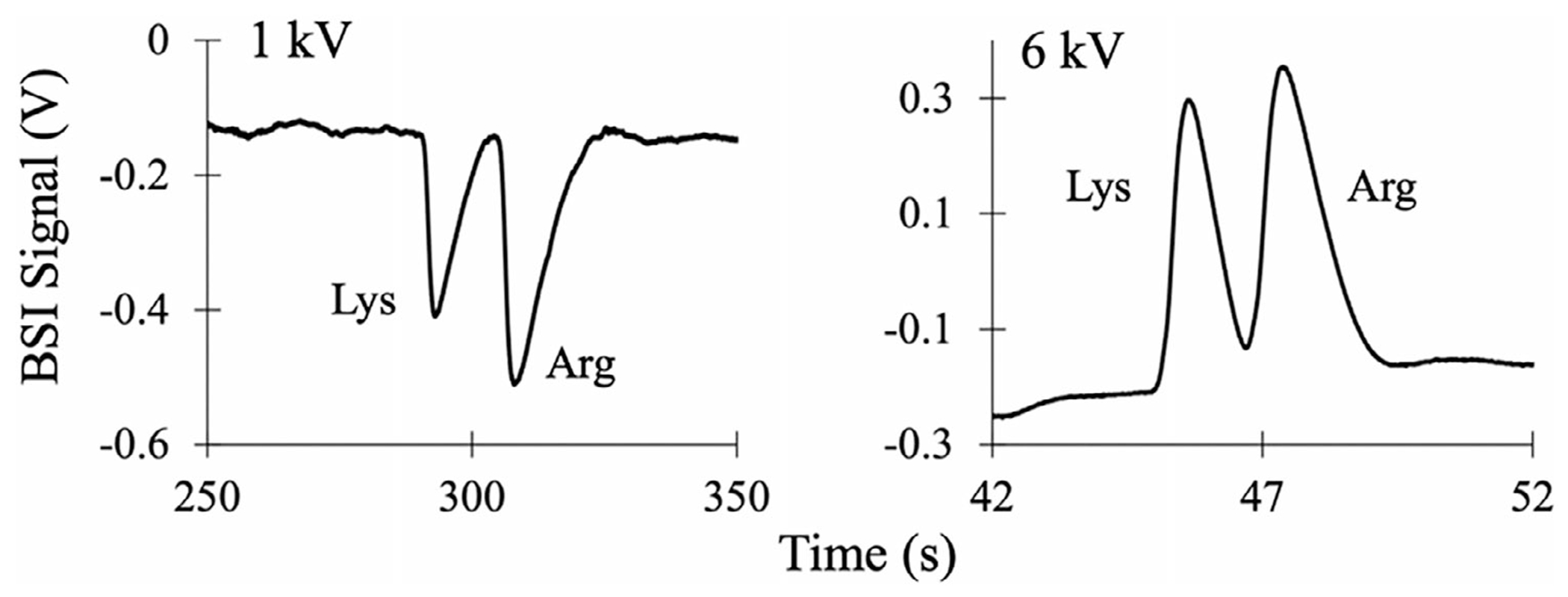
Electropherograms measured with BSI at 1 and 6 kV for the separation of Lys and Arg in a BGE of 4 M HAc at pH 2.1. Using BSI detection, the sign and amplitude of the BSI signal can change with separation voltage.

BSI detection, on the other hand, offers a distinct contrast to these traditional approaches. In BSI, the signal amplitude is influenced by the analyte concentration, the separation conditions, and the applied voltage, with the latter arising from conductivity differences between the analyte zone and the BGE [28]. Figure 1, for example, shows representative BSI electropherograms (*λ* = 647nm) for the separation of unlabeled Lys and Arg (1 mM each) in a 4 M acetic acid (HAc) BGE at pH 2.1. At a low separation voltage of 1 kV, the peaks have a negative polarity. When the separation voltage is increased to 6 kV, however, the peaks flip to positive-going with a larger amplitude [28].

In a previous report, we showed that analyte concentration, which affects both the refractive index and conductivity of the sample zone, leads to changes in the BSI signal with applied voltage [28]. The BSI signal can be described using Equation (3):

(3) $$\text{BSI Signal}=\frac{\partial n}{\partial C}\cdot C(x,t)+K^{\prime }\cdot J^{2}\cdot {\left(\frac{1}{\kappa_{x}(x,t)}\right)}^{2}\text{,}$$

where, *n* is the refractive index, *C* is the concentration, *K*’ is a proportionality constant, *J* is the current density, *κ_x_* and is the local conductivity. The first term in Equation (3) represents the RI contribution to the BSI signal, while the second term describes the voltage dependence. Previous studies have shown that the BSI signal increases linearly with the square of the separation voltage [17, 28]. This voltage dependence leads to the second term in Equation (3), which is written in terms of the conductivity of the analyte zone. Since the current density is proportional to the applied separation voltage, the second term becomes increasingly important as the separation voltage increases [28].

### 3.2 | BSI Signal Peak Narrowing with Voltage

The contribution to the BSI signal from each of the two terms in Equation (3) can work in the same direction or opposite to one another. In cases where the conductivity of the analyte zone is lower than the surrounding BGE, for example, the two terms in Equation (3) can act in opposition. This is the case for the separation shown in Figure 1. At low applied voltage, the first term in Equation (3) leads to negative-going BSI signals, which transition to positive-going peaks with increased amplitude as the voltage is increased and the second term becomes dominant. At the separation voltage where the peaks transition in sign, an interesting and significant narrowing of the peaks is observed.

**FIGURE 2 |:**
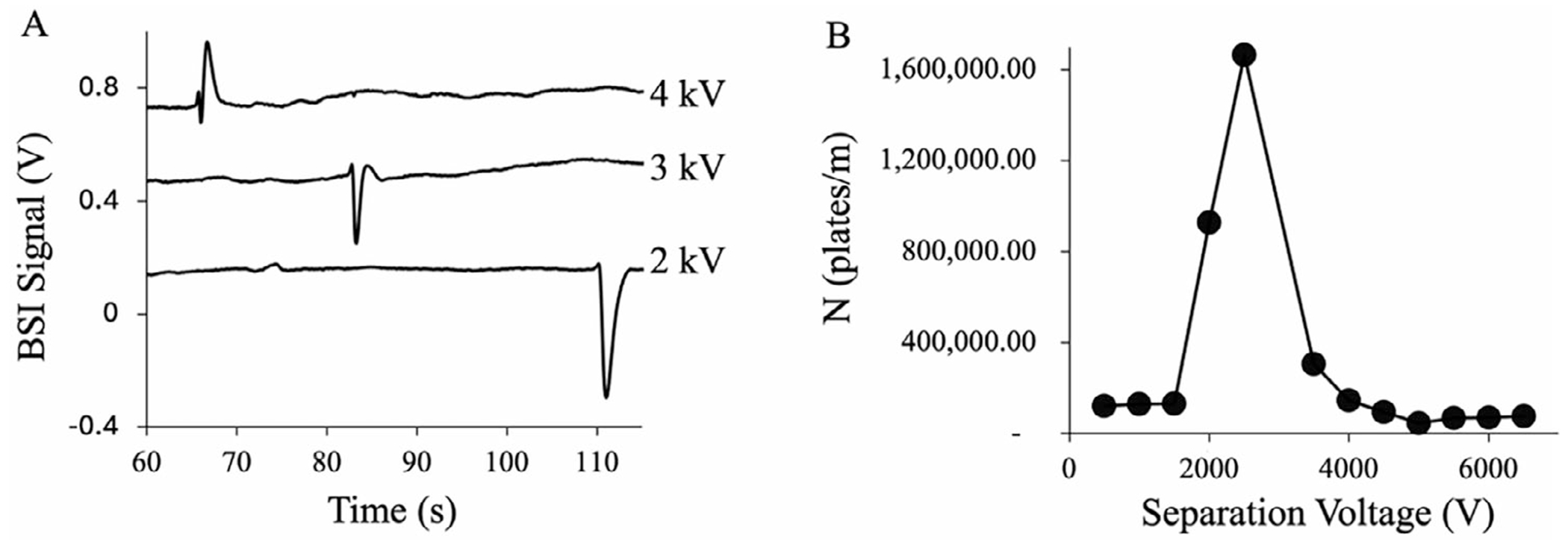
(A) Electropherograms for Arg as a function of separation voltage in a BGE of 4 M HAc at pH 2.1. As the separation voltage increases, the BSI signal for Arg narrows and changes polarity. (B) Apparent peak efficiency of Arg as a function of separation voltage.

The peak-narrowing can be seen in Figure 2A, which shows representative electropherograms of 1 mM Arg as a function of separation voltage. Using the same BGE of 4 M HAc at pH 2.1, the Arg signal is negative at low separation voltage (2 kV) and transitions to a positive signal at 4 kV. Near a separation voltage of 3 kV, as the BSI signal transitions from negative to positive going, a significant narrowing of the Arg peak is observed.

To evaluate the change in “apparent peak efficiency”, electropherograms were measured as the separation voltage was increased from 1 to 6.5 kV. The calculated apparent peak efficiencies (plates/m) for Arg as a function of separation voltage are plotted in Figure 2B. As shown in the plot, there is a significant increase in the apparent peak efficiency from 1.3 × 10^5^ to 1.6 × 10^6^ plates/m near the separation voltage that leads to the change in signal polarity. This is more than a 10-fold increase in *N* and suggests that the influence of voltage on the BSI signal can be strategically leveraged to improve apparent peak efficiencies and potentially resolution.

**FIGURE 3 |:**
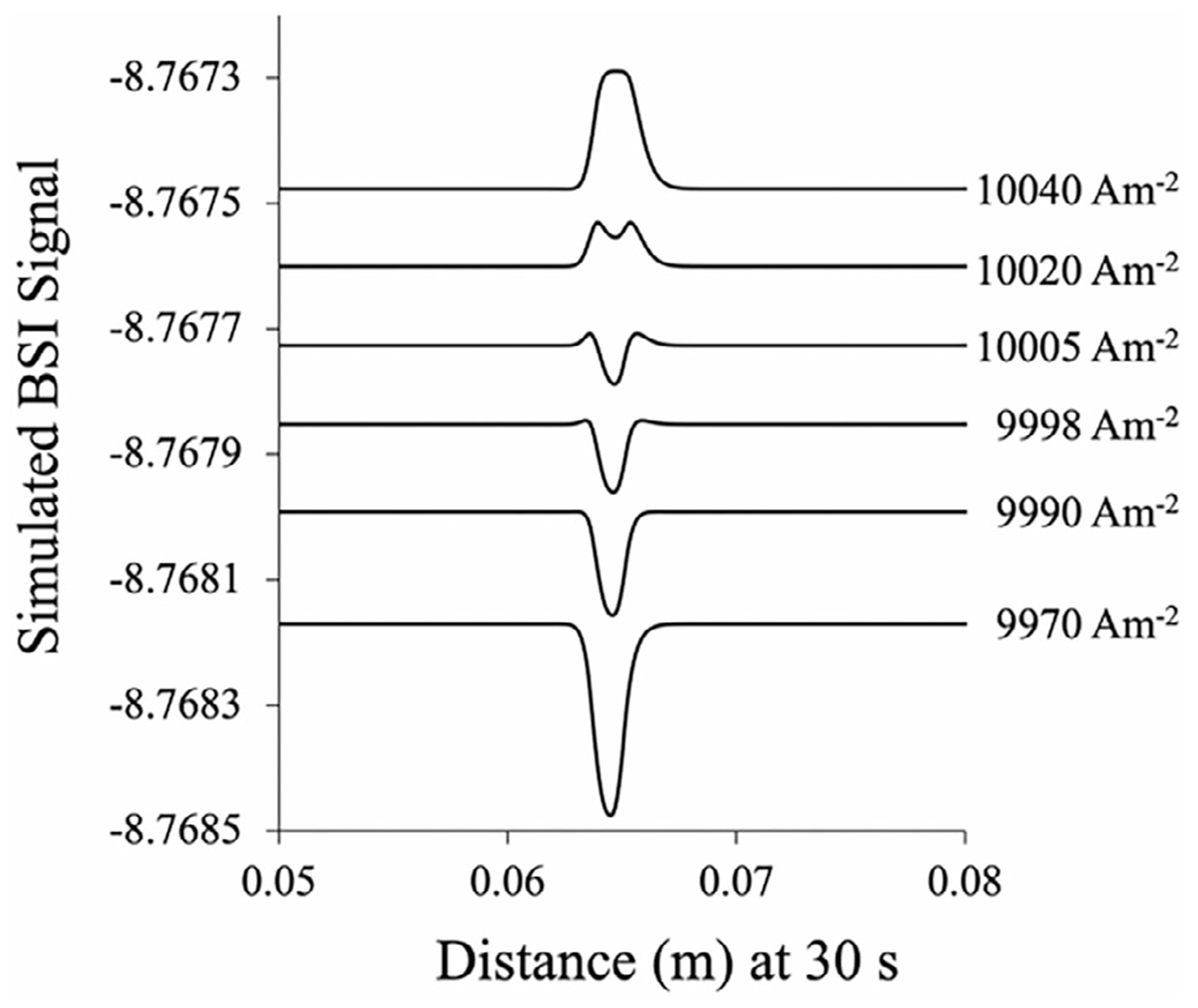
Simulations of the BSI signal for Arg in a BGE of 4 M HAc at pH 2.1. As the separation voltage (current density) increases, the peak changes polarity. A narrowing of the peak is observed in the BSI signal near the transition.

For BSI signals where the two terms in Equation (3) act in opposite directions, the resulting signal resembles the sum of two opposing functions—a Gaussian function representing the RI signal (first term) and a squared Gaussian function representing the conductivity-related term (second term). This is explored in Figure 3, where COMSOL simulations incorporating Equation (3) are used to simulate the BSI signal for 1 mM Arg in 4 M HAc as a function of separation voltage (current density). As the current density increases, the BSI signal for Arg changes sign in agreement with the measurements, as shown in Figure 2. A close inspection also reveals a narrowing of the Arg signal in the 9998 and 10 005 Am^−2^ simulated electropherograms, just before the signal changes sign. This is due to the nonlinear dependence of the BSI signal on the local conductivity (local field strength) and its linear dependence on the refractive index. When these two mechanisms contribute to the overall BSI signal in opposite directions, their sum will produce a resultant peak, which narrows near the transition. In other words, the BSI response is amplified near the center of the peak as the tails become suppressed. Interestingly, the simulations predict significantly less narrowing than measured experimentally, which appears to arise from the asymmetry of the measured peak shape, as opposed to the more symmetric peak shape used in the simulation.

### 3.3 | BSI Signal Peak Narrowing with Photothermal Excitation

**FIGURE 4 |:**
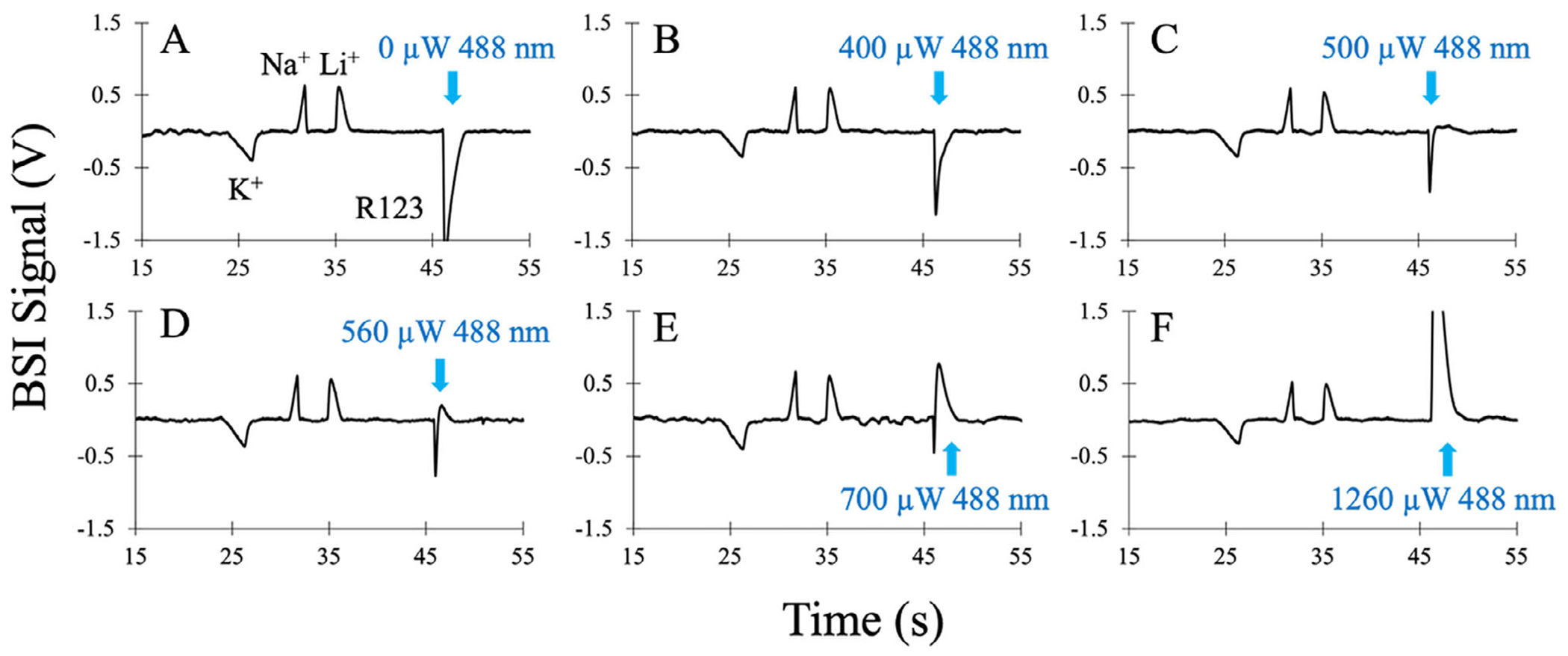
(A) Electropherogram for the separation of 50 μM each of K^+^, Na^+^, Li^+^, and 25 μM rhodamine 123 (R123) in a 20 mM imidazole buffer at pH 6.2. The peaks are detected using BSI at 543 nm using a separation voltage of 3000 V (300 V/cm). (B–F) Electropherograms measured using the same sample and separation conditions with the addition of photothermal excitation of R123 using coaxially delivered light at 488 nm at the indicated powers.

In addition to separation voltage, the BSI signal can also be enhanced by photothermal processes, as discussed previously [29]. This is illustrated in Figure 4, which shows a series of electropherograms of a sample mixture containing 50 μM each of K^+^, Na^+^, Li^+^, and 25 μM rhodamine 123 (R123). In all electropherograms, the mixture was separated in a BGE of 20 mM imidazole at pH 6.2 using a separation voltage of3 kV (300 V/m). For these measurements, the BSI signal was generated using a 543 nm HeNe light source focused into the detection volume. A second light source, the 488 nm line from an argon ion laser, was coaxially focused into the same detection zone to specifically excite R123, as previously described [29].

Figure 4A shows the BSI electropherogram for the four analytes in the absence of photothermal excitation with 488 nm light. The remaining BSI electropherograms in Figure 4B–F were measured at increasing levels of 488 nm excitation ranging from 400 to 1260 μW, respectively. As expected, the 488 nm excitation does not affect the BSI signal from the inorganic ions, and their peak shapes and amplitudes remain unchanged in the electropherograms shown in Figure 4. R123, on the other hand, strongly absorbs 488 nm excitation, which has a significant effect on its BSI signal.

**FIGURE 5 |:**
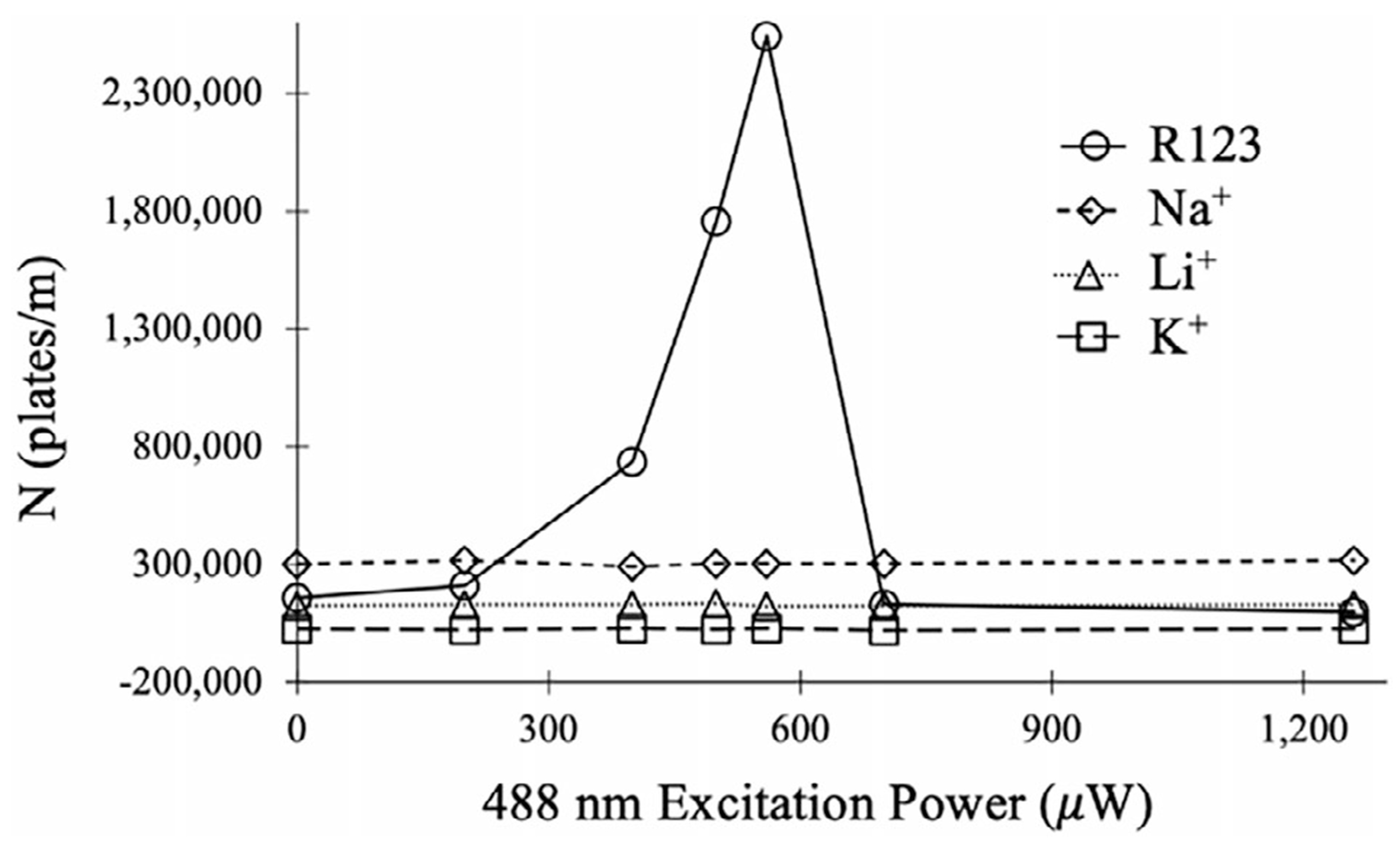
Calculated peak efficiencies for the data shown in Figure 4.

In the absence of 488 nm excitation, the R123 peak exhibits a large, negative amplitude peak in the BSI signal (Figure 4A). With increasing levels of 488 nm excitation power (Figure 4B–F), the amplitude of the R123 peak reduces toward the baseline and eventually changes sign and grows in the positive signal direction. As discussed previously [29], nonradiative relaxation of excited R123 releases heat into the local surroundings, which modifies both the RI and viscosity (conductivity) of the analyte zone [31, 32, 30, 40]. Since both RI and conductivity influence the BSI signal (Equation 3), photothermal excitation can strongly enhance the BSI signal and extend analyte detection down to the submicromolar level [29]. Photothermal excitation can also lead to changes in peak polarity, as shown in Figure 4. Like that shown for the separation voltage measurements in Figure 2, the transition in R123 peak polarity in Figure 4 is accompanied by a significant narrowing of the peak and an increase in apparent peak efficiency. Calculated peak efficiencies (plates/m) for the four analytes in Figure 4 at the indicated 488 nm power levels are plotted in Figure 5.

As shown in Figure 5, peak efficiencies for Na^+^, K^+^, and Li^+^ remain approximately constant at all power levels since they do not absorb 488 nm light. The constant values also suggest that nonresonant heating of the sample zone from the 488 nm laser is insignificant. The data for R123, on the other hand, show a sharp rise in peak efficiency with 488 nm power that peaks near 560 μW. As evident in Figure 4, this is the power required to position the peak just before its change in polarity. The apparent plate numbers for R123 increase from 1.6 × 10^5^ plates/m in the absence of photothermal excitation to over 2.5 × 10^6^ plates/m at 560 μW of 488 nm excitation, representing a ~16-fold improvement in apparent peak efficiency. These findings demonstrate that by coupling BSI detection with photothermal excitation, it is possible to selectively enhance the apparent peak efficiency of specific analytes.

### 3.4 | Resolution Increase with Photothermal Excitation

**FIGURE 6 |:**
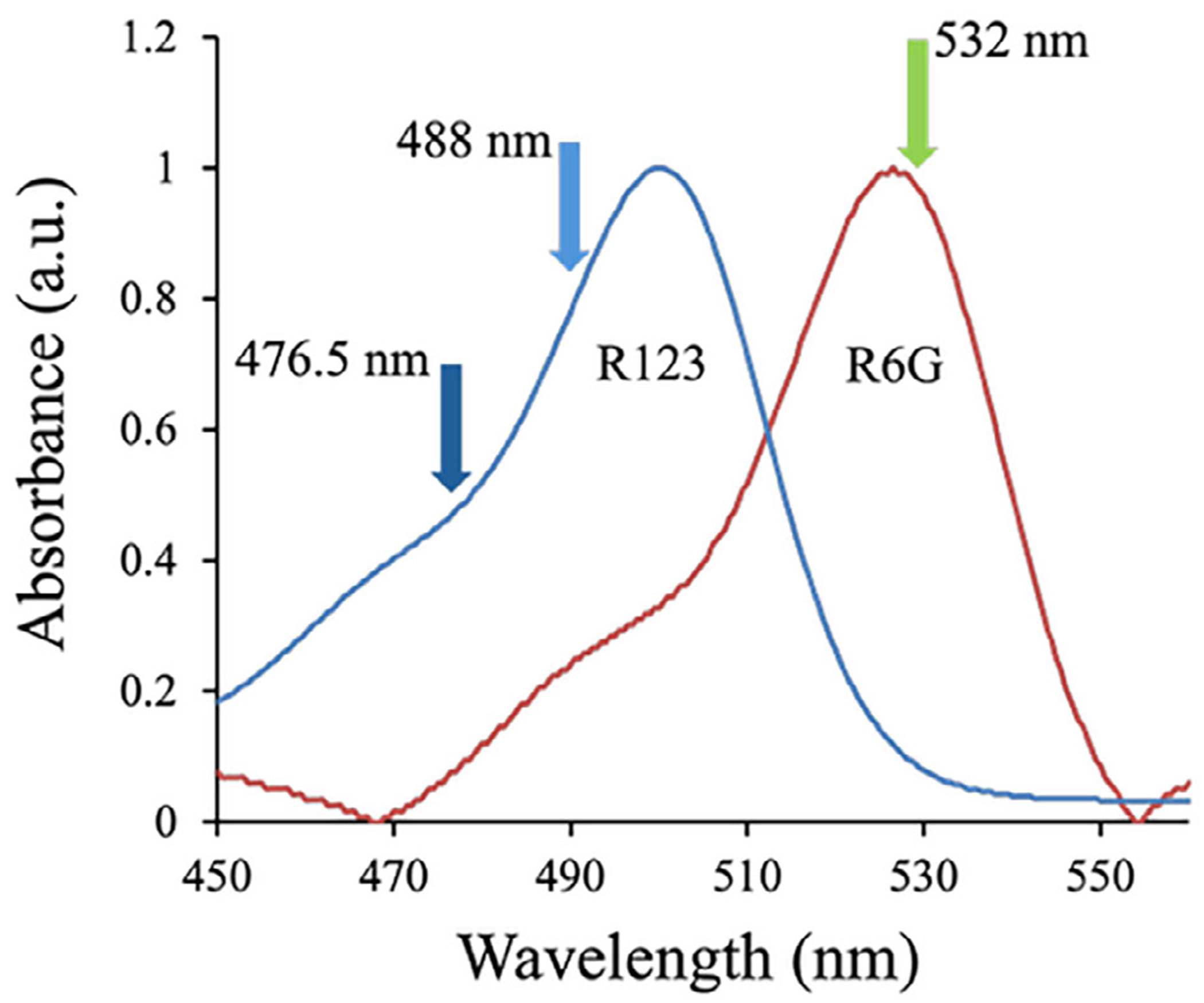
Absorption spectra of R123 and R6G with arrows denoting the wavelengths used for photothermal excitation.

While high peak efficiency is desirable and both the voltage-sensitive mechanism and photothermal approach can lead to over an order of magnitude improvement, the more important question is whether the improved efficiency translates to better resolution. To test this using the photothermal approach, the previous sample mixture (50 μM of Na^+^, K^+^, and Li^+^ with 25 μM of R123) was spiked with 25 μM of rhodamine 6G (R6G). R123 and R6G have similar electrophoretic mobilities under these conditions, but their spectra have minimal overlap (Figure 6), which enables preferential excitation. For these experiments, the BSI signal was measured using 633 nm light that is not resonant with either dye.

**FIGURE 7 |:**
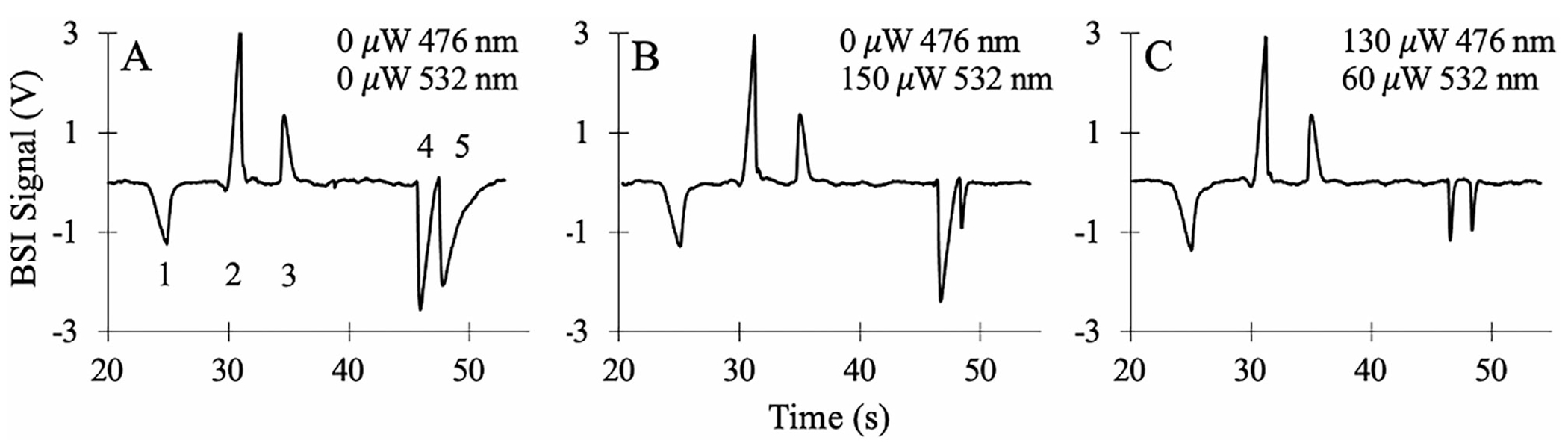
(A) Electropherogram of a sample consisting of 50 μM each of K^+^ (1), Na^+^ (2), Li^+^ (3), 25 μM R123 (4), and 25 μM R6G (5) separated in a20 mM imidazole buffer atpH 6.2. The peaks are detected using BSI at 633 nm, and the separation voltage is 3000 V (300 V/cm). (B) Electropherogram of the same sample using the same separation conditions as in panel A, but with specific photothermal excitation of R6G (5) using 150 μW of 532 nm light. (C) Photothermal excitation of both R123 (130 μW of 476.5 nm light) and R6G (60 μW of 532 nm light) to narrow the peaks and optimize resolution.

Figure 7A shows the separation of the five analytes (Li^+^, Na^+^, K^+^, R123, and R6G) detected with BSI at 633 nm. As before, the sample was separated in a 20 mM imidazole buffer at pH 6.2 using a separation voltage of 3000 V (300 V/m). In the absence of photothermal excitation (Figure 7A), both dye molecules appear as negative-going peaks in the BSI signal. To preferentially excite R123 and R65, the 476.5 nm line from an argon ion laser and the 532 nm line from a diode laser, respectively, were coupled into the same fiber optic used to deliver the 633 nm light for BSI detection. This arrangement results in coaxial photothermal excitation in the detection volume of the capillary as previously described [29]. As shown in Figure 6, these lines will preferentially but not exclusively excite each dye.

Figure 7B shows the measured electropherogram using preferential photothermal excitation of R6G with 150 μW of 532 nm to selectively narrow the peak. While the apparent peak efficiency of the R6G peak has been significantly increased, the R123 peak remains unaffected by the 532 nm excitation. Figure 7C shows the results of adding 130 μW of 476.5 nm excitation to narrow the R123 BSI signal. Since 476.5 nm excitation still excites R6G somewhat (Figure 6), the 532 nm light was reduced to 60 μW to optimize the peaks for both dyes. Figure 7C shows that the R123 and R6G peaks can be simultaneously narrowed by optimizing the specific photothermal excitation. The apparent peak efficiency for R123 improves from 2.2 × 10^5^ (Figure 7A) to 2.1 × 10^6^ N/m (Figure 7C) with selective excitation, while R6G improves from 9.7 × 10^4^ to 2.2 × 10^6^ N/m. Comparing Figure 7C with Figure 7A, moreover, clearly shows an improvement in resolution. The calculated resolution between R123 and R6G improves from *R* = 1.1 in the absence of photothermal excitation (Figure 7A) to *R* = 5.3 (Figure 7C) upon optimizing photothermal excitation.

Since both dyes are fluorescent, we can further validate that the narrowing mechanism is unique to the BSI signal by simultaneously measuring the fluorescence electropherogram. As reported previously, the BSI and photothermal light sources are delivered to the detection volume using the same optical fiber and focusing optics [29]. The shared optical path results in simultaneously measured BSI and fluorescence signals that are perfectly registered, enabling direct comparisons between electropherograms. For these measurements, a single photothermal excitation line at 488 nm was used to excite both dyes (see Figure 6).

**FIGURE 8 |:**
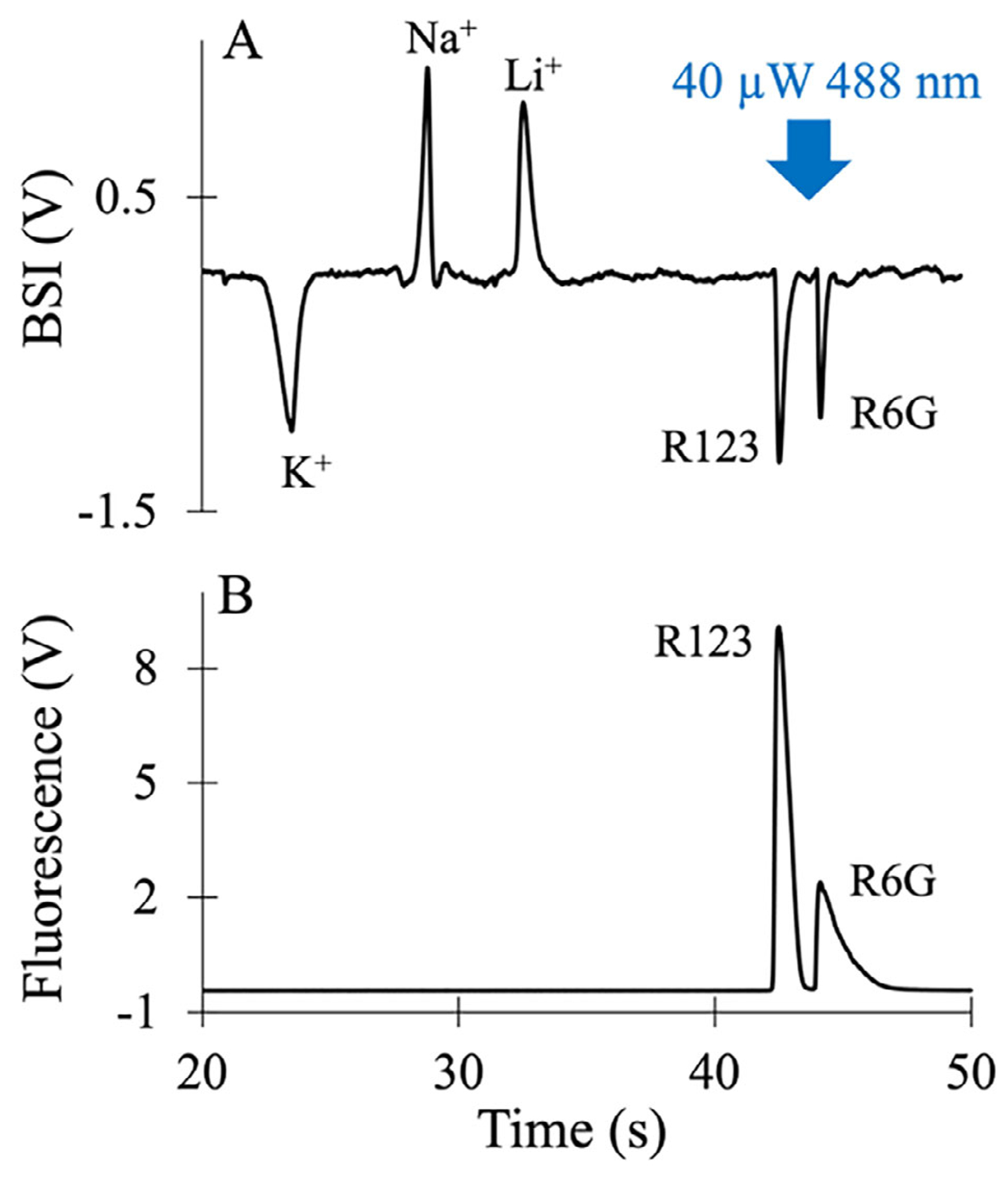
Simultaneously measured BSI (633 nm) and fluorescence electropherograms for the separation of 50 μM each of K+, Na+, Li+, and 25 μM each of R123 and R6G in 20 mM imidazole buffer at pH 6.2. Excitation of R123 and R6G using 40 μW of 488 nm light (see Figure 6) is used to both narrow the BSI signals using the photothermal mechanism and generate the fluorescence electropherogram.

Figure 8 shows the simultaneously measured BSI and fluorescence electropherograms for the same sample mixture used in Figure 7. The 488 nm power level was adjusted to optimize the peak efficiency of the R6G peak, which resulted in the R123 peak being narrowed but not optimized. Still, the BSI electropherogram clearly shows significantly sharper peaks for the two dyes when compared with the simultaneously collected fluorescence electropherogram. The peak efficiencies of R123 and R6G are 9.4 × 10^4^ and 1.8 × 10^5^ N/m, respectively, in the BSI electropherogram and 2.9 × 10^4^ and 1.5 × 10^4^ N/m, respectively, in the fluorescence electropherogram. This leads to a resolution in the BSI electropherogram of *R* = 2.6 compared with *R* = 1.0 in the fluorescence electropherogram. The comparison in Figure 8 confirms that the observed peak-narrowing arises from modifications in the BSI signal and not the analyte zones themselves, justifying the use of the term “apparent peak efficiency”. It also shows that photothermal BSI can be tuned to outperform traditional fluorescence detection in resolving closely migrating analytes if the analytes have the appropriate spectral properties.

### 3.5 | Resolution Increase with Separation Voltage

**FIGURE 9 |:**
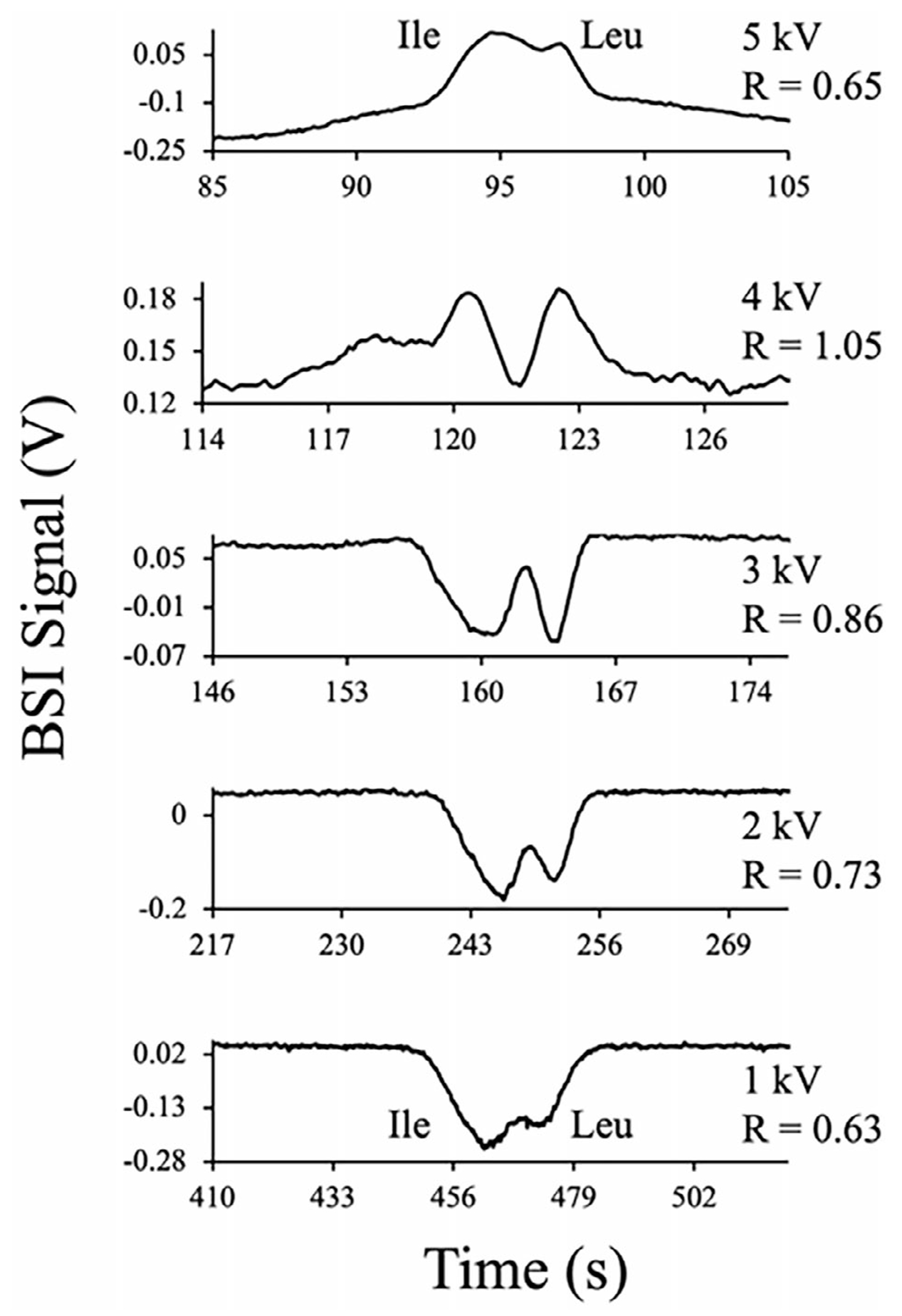
BSI electropherograms for the separation of the amino acid isomers leucine (Leu) and isoleucine (Ile) in 4 M HAc at pH 2.1 as a function of separation voltage. Resolution (R) between the peaks is improved at separation voltages near the change in sign.

Finally, we show that the voltage-dependent BSI narrowing mechanism also leads to enhanced resolution, providing another approach for resolving analytes that are difficult to separate. Resolving isomeric compounds such as the amino acids leucine (Leu) and isoleucine (Ile) often presents challenges given their similar structures. These amino acids have important applications in the diagnosis of metabolic disorders like maple syrup urine disease and for evaluating the risk and progression of some cancers. Figure 9 shows a series of electropherograms for the separation of Leu and Ile measured as a function of separation voltage. The amino acids (1 mM each) were separated in a BGE of 4 M HAc at pH 2.1 using separation voltages ranging from 1 to 5 kV as indicated in Figure 9.

At both low (1 kV) and high separation voltages (5 kV), the Leu and Ile zones co-migrate, leading to significantly overlapped peaks in the BSI signal with *R* values near 0.6. In the intermediary voltages of 2 to 4 kV, where the peaks transition from negative to positive polarity, resolution is improved as reflected in the *R* values. While it is difficult to assess the peak shape correctly due to the baseline variation, the highest resolution of the set was recorded for 4 kV. This is consistent with the increase in peak efficiency observed previously in the voltage-dependent mechanism (Figure 2). The results demonstrate that BSI, when combined with voltage tuning or photothermal excitation, offers a powerful and selective method for enhancing peak efficiency and resolution in CE.

## 4 | Concluding Remarks

The versatility and tunability of BSI as a detection modality in CE are explored to improve apparent peak efficiency and resolution. Since the BSI signal is sensitive to both analyte concentration (RI) and local conductivity, it presents unique opportunities for tuning the signal using external parameters such as the separation voltage and photothermal excitation. We show, for example, that both mechanisms can lead to a change in signal polarity and that the change in polarity is accompanied by a significant increase in apparent peak efficiency near the transition. In the voltage-dependent mechanism, the conductivity sensitivity of the BSI signal leads to peak narrowing when the separation voltage is tuned to coincide with the peak transition. Since analytes with similar mobilities (conductivities) will invert near the same separation voltage, this approach is optimized to resolve structurally similar analytes like isomers, which is demonstrated in the improved resolution for the separation of Leu and Ile.

Similarly, photothermal excitation can lead to polarity changes in the BSI signal through local heating, which influences both the refractive index and conductivity of the analyte zone. This mechanism also leads to an increase in the apparent peak efficiency near the peak transition. This is demonstrated by selectively exciting two dye molecules that migrate closely in the electropherogram, optimizing their apparent peak efficiencies for increased separation resolution. For analytes with regions of nonoverlapping absorption spectra, this mechanism provides a means of selectively and independently tuning the efficiency of each peak for optimal resolution. Together, these results highlight unique aspects of the BSI signal that can lead to new avenues for probing complex mixtures.

## Data Availability

The data that support the findings of this study are available from the corresponding author upon reasonable request.

## Abbreviations:

BGE: background electrolyte
BSI: Backscatter Interferometry
PCE: planar capillary electrophoresis
R123: rhodamine 123
R6G: rhodamine 6G
RI: refractive index

## References

[1] Bowser MT, Bebault GM, Peng X, and Chen DD, “Redefining the Separation Factor: A Potential Pathway to a Unified Separation Science,” Electrophoresis 18, no. 15 (1997): 2928–2934, 10.1002/elps.1150181534. 9504832

[2] Craig LC and Post O, “Apparatus for Countercurrent Distribution,” Analytical Chemistry 21, no. 4 (1949): 500–504, 10.1021/ac60028a013.

[3] Martin AJP and Synge RLM, “A New Form of Chromatogram Employing Two Liquid Phases: A Theory of Chromatography. 2. Application to the Micro-Determination of the Higher Monoamino-Acids in Proteins,” Biochemical Journal 35, no. 12 (1941): 1358–1368, 10.1042/bj0351358. 16747422 PMC1265645

[4] Jorgenson JW and Lukacs KD, “Zone Electrophoresis in Open-Tubular Glass Capillaries,” Analytical Chemistry 53, no. 8 (1981): 1298–1302, 10.1021/ac00231a037. 7261333

[5] Giddings JC, “Generation of Variance, Theoretical Plates, Resolution, and Peak Capacity in Electrophoresis and Sedimentation,” Separation Science 4, no. 3 (1969): 181, 10.1080/01496396908052249.

[6] Zhao C, Zhang W, and Yang C, “Dynamic Electroosmotic Flows of Power-Law Fluids in Rectangular Microchannels,” Micromachines 8, no. 2 (2017).

[7] Wätzig H and Günter S, “Capillary Electrophoresis—A High Performance Analytical Separation Technique,” Clinical Chemistry & Laboratory Medicine 41, no. 6 (2003): 724–738. 12880135 10.1515/CCLM.2003.112

[8] Woods LA, Roddy TP, Paxon TL, and Ewing AG, “Electrophoresis in Nanometer Inner Diameter Capillaries with Electrochemical Detection,” Analytical Chemistry 73, no. 15 (2001): 3687–3690, 10.1021/ac010053e. 11510835

[9] Ghosal S, “Band Broadening in a Microcapillary with a Stepwise Change in the ζ-potential,” Analytical Chemistry 74, no. 16 (2002): 4198–4203, 10.1021/ac025630t. 12199593

[10] Schure MR and Lenhoff AM, “Consequences of Wall Adsorption in Capillary Electrophoresis: Theory and Simulation,” Analytical Chemistry 65, no. 21 (1993): 3024–3037, 10.1021/ac00069a015.

[11] Mai TD and Hauser PC, “Study on the Interrelated Effects of Capillary Diameter, Background Electrolyte Concentration, and Flow Rate in Pressure Assisted Capillary Electrophoresis with Contactless Conductivity Detection,” Electrophoresis 34, no. 12 (2013): 1796–1803, 10.1002/elps.201200586. 23417350

[12] Hjertén S, “Zone Broadening in Electrophoresis with Special Reference to High-Performance Electrophoresis in Capillaries: An Interplay between Theory and Practice,” Electrophoresis 11, no. 9 (1990): 665–690. 2257839 10.1002/elps.1150110904

[13] Gaš B, Štědrý M, and Kenndler E, “Peak Broadening in Capillary Zone Electrophoresis,” Electrophoresis 18, no. 12-13 (1997): 2123–2133. 9456027 10.1002/elps.1150181203

[14] Kubáň P and Hauser PC, “Effects of the Cell Geometry and Operating Parameters on the Performance of an External Contactless Conductivity Detector for Microchip Electrophoresis,” Lab on a Chip 5, no. 4 (2005): 407–415. 15791338 10.1039/b418845d

[15] Poulsen NN, Østergaard J, Petersen NJ, , “Automated Coating Procedures to Produce Poly(ethylene glycol) Brushes in Fused-Silica Capillaries,” Journal of Separation Science 40, no. 3 (2017): 779–788, 10.1002/jssc.201600878. 27868374

[16] Gaš B and Kenndler E, “Dispersive Phenomena in Electromigration Separation Methods,” Electrophoresis 21, no. 18 (2000): 3888–3897. 11192113 10.1002/1522-2683(200012)21:18<3888::AID-ELPS3888>3.0.CO;2-D

[17] Dunn RC, “High-Speed Capillary Electrophoresis Using a Thin-Wall Fused-Silica Capillary Combined with Backscatter Interferometry,” Analytical Chemistry 92, no. 11 (2020): 7540–7546, 10.1021/acs.analchem.9b05881. 32352792

[18] Culbertson CT and Jorgenson JW, “Increasing the Resolving Power of Capillary Electrophoresis through Electroosmotic Flow Control Using Radial Fields,” Journal of Microcolumn Separations 11, no. 3 (1999): 167–174, 10.1002/(SICI)1520-667X(1999)11:3<167::AID-MCS1>3.0.CO;2-Y.

[19] De Silva M, Opallage PM, and Dunn RC, “Investigation of Induced Electroosmotic Flow in Small-Scale Capillary Electrophoresis Devices: Strategies for Control and Reversal,” Electrophoresis 45, no. 19-20 (2024): 1764–1774, 10.1002/elps.202400107. 39054801 PMC11502244

[20] Gillogly JA and Lunte CE, “pH-mediated Acid Stacking with Reverse Pressure for the Analysis of Cationic Pharmaceuticals in Capillary Electrophoresis,” Electrophoresis 26, no. 3 (2005): 633–639, 10.1002/elps.200410061. 15690436 PMC2519829

[21] Chang Y-S, Shih C-M, Li Y-C, and Lin C-H, “Large-Volume Sample Sweeping with a High Theoretical Plate Number Using a Coupled-Capillary in Capillary Electrophoresis,” Analytical Sciences 22, no. 4 (2006): 557–561, 10.2116/analsci.22.557. 16760597

[22] Wang Z, Swinney K, and Bornhop DJ, “Attomole Sensitivity for Unlabeled Proteins and Polypeptides with On-Chip Capillary Electrophoresis and Universal Detection by Interferometric Backscatter,” Electrophoresis 24, no. 5 (2003): 865–873, 10.1002/elps.200390109. 12627449

[23] De Silva M, Opallage PM, and Dunn RC, “Direct Detection of Inorganic Ions and Underivatized Amino Acids in Seconds Using High-Speed Capillary Electrophoresis Coupled with Back-Scatter Interferometry,” Analytical Methods 13, no. 11 (2021): 1340–1348, 10.1039/D0AY02218G. 33491683

[24] Swinney K, Pennington J, and Bornhop DJ, “Universal Detection in Capillary Electrophoresis with a Micro-Interferometric Backscatter Detector,” Analyst 124, no. 3 (1999): 221–225, 10.1039/a809691k.

[25] Dunn RC, “Wavelength Modulated Back-Scatter Interferometry for Universal, On-Column Refractive Index Detection in Picoliter Volumes,” Analytical Chemistry 90, no. 11 (2018): 6789–6795, 10.1021/acs.analchem.8b00771. 29762009

[26] Dunn RC, “Compact, Inexpensive Refractive Index Detection in Femtoliter Volumes Using Commercial Optical Pickup Technology,” Analytical Methods 11, no. 17 (2019): 2303–2310, 10.1039/C9AY00369J.

[27] Mulkerns NMC, Hoffmann WH, Lindsay ID, and Gersen H, “An Analysis of Semicircular Channel Backscattering Interferometry through Ray Tracing Simulations,” Sensors-Basel 22, no. 11 (2022): 4301, 10.3390/s22114301. 35684929 PMC9185450

[28] De Silva M and Dunn RC, “Electric Field-enhanced Backscatter Interferometry Detection for Capillary Electrophoresis,” Scientific Reports 14, no. 1 (2024): 2110, 10.1038/s41598-024-52621-3. 38267528 PMC10808210

[29] Opallage PM, De Silva M, Kariuki SM, Raheel AA, and Dunn RC, “Photothermal Backscatter Interferometry for Enhanced Detection in Capillary Electrophoresis,” Analytical Chemistry 96, no. 32 (2024): 13234–13241, 10.1021/acs.analchem.4c02312. 39072412

[30] Dovichi NJ and Harris JM, “Laser-Induced Thermal Lens Effect for Calorimetric Trace Analysis,” Analytical Chemistry 51, no. 6 (1979): 728–731, 10.1021/ac50042a034.

[31] Dovichi NJ, Nolan TG, and Weimer WA, “Theory for Laser-Induced Photothermal Refraction,” Analytical Chemistry 56, no. 9 (1984): 1700–1704, 10.1021/ac00273a038.

[32] Nolan TG, Weimer WA, and Dovichi NJ, “Laser-induced Photothermal Refraction for Small Volume Absorbance Determination,” Analytical Chemistry 56, no. 9 (1984): 1704–1707, 10.1021/ac00273a039.

[33] Cassano CL, Mawatari K, Kitamori T, and Fan ZH, “Thermal Lens Microscopy as a Detector in Microdevices,” Electrophoresis 35, no. 16 (2014): 2279–2291, 10.1002/elps.201300430. 24435958

[34] Bruno AE, Paulus A, and Bornhop DJ, “Thermooptic Absorption Detection in 25-Mu-M-ID Capillaries—Capillary Electrophoresis of Dansyl-Amino Acids Mixtures,” Applied Spectroscopy 45, no. 3 (1991): 462–467, 10.1366/0003702914337371.

[35] Betzig E, Patterson GH, Sougrat R, , “Imaging Intracellular Fluorescent Proteins at Nanometer Resolution,” Science 313, no. 5793 (2006): 1642–1645, 10.1126/science.1127344. 16902090

[36] Hell SW and Wichmann J, “Breaking the Diffraction Resolution Limit by Stimulated Emission: Stimulated-Emission-Depletion Fluorescence Microscopy,” Optics Letters 19, no. 11 (1994): 780–782, 10.1364/OL.19.000780. 19844443

[37] Klar TA, Engel E, and Hell SW, “Breaking Abbe’s Diffraction Resolution Limit in Fluorescence Microscopy with Stimulated Emission Depletion Beams of Various Shapes,” Physical Review E, Statistical, Nonlinear, and Soft Matter Physics 64, no. 6 Pt 2 (2001): 066613, 10.1103/PhysRevE.64.066613. 11736302

[38] Rust MJ, Bates M, and Zhuang X, “Sub-diffraction-limit Imaging by Stochastic Optical Reconstruction Microscopy (STORM),” Nature Methods 3, no. 10 (2006): 793–795, 10.1038/nmeth929. 16896339 PMC2700296

[39] Dubsky P, Ordogova M, Maly M, and CEval RM, “All-in-One Software for Data Processing and Statistical Evaluations in Affinity Capillary Electrophoresis,” Journal of Chromatography A 1445 (2016): 158–165, 10.1016/j.chroma.2016.04.004. 27062723

[40] Bruno AE, Paulus A, and Bornhop DJ, “Thermo-Optical Absorption Detection in 25-μm-i.d. Capillaries: Capillary Electrophoresis of Dansyl-Amino Acids Mixtures,” Applied Spectroscopy 45, no. 3 (1991): 462–467, 10.1366/0003702914337371.

